# Green Tea Polyphenols, Mimicking the Effects of Dietary Restriction, Ameliorate High-Fat Diet-Induced Kidney Injury via Regulating Autophagy Flux

**DOI:** 10.3390/nu9050497

**Published:** 2017-05-14

**Authors:** Xiao Xie, Weijie Yi, Piwei Zhang, Nannan Wu, Qiaoqiao Yan, Hui Yang, Chong Tian, Siyun Xiang, Miying Du, Eskedar Getachew Assefa, Xuezhi Zuo, Chenjiang Ying

**Affiliations:** 1 Department of Nutrition and Food Hygiene, School of Public Health, Tongji Medical College, Huazhong University of Science and Technology, Wuhan 430030, Hubei, China; xxiao31933@163.com (X.X.); yiweijie2014@126.com (W.Y.); m15927511308@163.com (N.W.); sy_xiang@hust.edu.cn (S.X.); eskedargat@yahoo.com (E.G.A); 2 MOE Key Lab of Environment and Health, School of Public Health, Tongji Medical College, Huazhong University of Science and Technology, Wuhan 430030, Hubei, China; 3 Department of Nutrition and Food Hygiene, School of Public Health and Management, Binzhou Medical University, Yantai 264003, Shandong, China; 4 Department of Clinical Nutrition, Tongji Hospital, Huazhong University of Science and Technology, Wuhan 430030, Hubei, China; zpwyipdnfk@163.com; 5 School of Public Health and Management, Wenzhou Medical University, Wenzhou 325003, Zhejiang, China; 15293590501@163.com; 6 Kecheng People’s Hospital, Quzhou 324000, Zhejiang, China; qsdyh@163.com; 7 School of Nursing, Tongji Medical College, Huazhong University of Science and Technology, Wuhan 430030, Hubei, China; tianchong0826@hust.edu.cn; 8 Department of Hotel Management, Tourism University, Guilin 541006, Guangxi, China; dumiying0914@gltu.cn

**Keywords:** green tea polyphenols, renal function, autophagy, high-fat diet, dietary restriction

## Abstract

Epidemiological and experimental studies reveal that Western dietary patterns contribute to chronic kidney disease, whereas dietary restriction (DR) or dietary polyphenols such as green tea polyphenols (GTPs) can ameliorate the progression of kidney injury. This study aimed to investigate the renal protective effects of GTPs and explore the underlying mechanisms. Sixty Wistar rats were randomly divided into 6 groups: standard diet (STD), DR, high-fat diet (HFD), and three diets plus 200 mg/kg(bw)/day GTPs, respectively. After 18 weeks, HFD group exhibited renal injuries by increased serum cystatin C levels and urinary *N*-acetyl-β-d-glucosaminidase activity, which can be ameliorated by GTPs. Meanwhile, autophagy impairment as denoted by autophagy-lysosome related proteins, including LC3-II, Beclin-1, p62, cathepsin B, cathepsin D and LAMP-1, was observed in HFD group, whereas DR or GTPs promoted renal autophagy activities and GTPs ameliorated HFD-induced autophagy impairment. In vitro, autophagy flux suppression was detected in palmitic acid (PA)-treated human proximal tubular epithelial cells (HK-2), which was ameliorated by epigallocatechin-3-gallate (EGCG). Furthermore, GTPs (or EGCG) elevated phosphorylation of AMP-activated protein kinase in the kidneys of HFD-treated rats and in PA-treated HK-2 cells. These findings revealed that GTPs mimic the effects of DR to induce autophagy and exert a renal protective effect by alleviating HFD-induced autophagy suppression.

## 1. Introduction

Chronic kidney disease (CKD), characterized by progressive decline in renal function, is very common in developed countries such as Norway (10.2%) and the USA (13.0%) [1]. Western dietary patterns, which are characterized by ingesting higher red and processed meats, saturated fats and sugars, are a major risk factor for metabolic disturbances including type 2 diabetes, cardiovascular disease, and kidney diseases [2]. Evidence from the Nurses’ Health Study showed that Western dietary patterns were associated with a higher odds ratio for urine albumin excretion and an elevated risk for rapid decline in estimated glomerular filtration rate (eGFR) [3]. A cross-sectional survey in 2001 and 2002 reported that the overall prevalence of CKD in China was 2.53% [4], but a national survey in China from 2007 to 2010 revealed that the prevalence of CKD was 10.8%. However, the prevalence of stage 3 and stage 4 CKD in China was still lower than that in developed countries. For example, the prevalence of stage 3 CKD was 1.6% in China, compared with 4.2% in Norway and 7.7% in the USA [1]. There are many factors associated with this change. For example, environmental factors and low birth weight. Besides, the westernization of diet in China in the past decades might also contribute to the increased prevalence of CKD in China. In vivo, it has been reported that high-fat diet (fat mainly from lard) consumption led to increased kidney weight, proteinuria, and renal hypertrophy, indicating that high-fat diet consumption causes functional and structural damages [5,6].

On the contrary, dietary restriction (DR), reduction of particular or total nutrient and energy intake without causing malnutrition (e.g., intermittent fasting, calorie restriction (CR), low calorie diet), has been reported to generate renal protective effect [7,8,9]. However, as the exact mechanism of DR is yet largely unknown, increased autophagy is suggested to be one of the possible routes. A growing body of recent evidence has implicated the importance of autophagy in both the maintenance of kidney homeostasis and disease pathogenesis [10,11]. For example, podocytes exhibit a high level of basal autophagy that could serve as a mechanism for their maintenance of cellular homeostasis [11]. In the kidneys of mice deficient in Beclin-1, there was significantly increased collagen deposition, and podocyte-specific Atg5 knockout or Atg5 deficiency in all tubule segments caused impairment of kidney function [10]. And autophagy suppressions were observed in the kidneys of patients with proteinuria or diabetic nephropathy (DN) [12,13]. Lysosomal process is crucial for the clearance of long-lived proteins and impaired organelles via autophagic degradation [14,15]. This function is dependent on lysosomal proteases, most importantly cathepsins, which contribute to the completed autophagic flux [16]. DR is considered as an effective strategy to induce autophagy. Kanamori et al. reported that starvation periods over 12 h led to robust autophagic vacuole stimulation and increased the expression of downstream lysosomal enzymes, such as cathepsin D, in the heart of mice, suggesting an enhancement of autophagic flux [17]. And DR resulted in complete improvement of diabetes-induced renal functional and histological abnormalities through regulating autophagy in diabetic Wistar fatty (fa/fa) rats [18].

Tea is a widely-consumed beverage around the world. Emerging studies have suggested that green tea polyphenols (GTPs) have strong protective effects against cardiovascular disease, metabolic syndrome, neurodegenerative diseases, cancer and renal injury [19,20,21,22]. Follow-up studies show that green tea consumption is associated with reduced total mortality and mortality due to cardiovascular diseases [23,24]. In diabetic nephropathy rat, GTPs could improve renal function and prevent glycogen accumulation in the renal tubules [25]. Epigallocatechin-3-gallate (EGCG), the most abundant constituent of GTPs, was shown to induce autophagy in recent studies in vitro. It has been reported that EGCG activated the autophagic pathways by inducing SIRT1, and had protective effects against human prion protein-induced neuronal cell toxicity [26]. And our previous study demonstrated that GTPs promote autophagy and alleviate high glucose-induced inhibition of autophagy in endothelial cells [27]. Notably, GTPs has been shown to be an inducer of autophagy in several cell culture models. However, few studies have investigated GTPs to induce autophagy in vivo. 

As GTPs exert an autophagy-inducing effect just like DR, we hypothesize that GTPs may protect against HFD-induced renal function impairment by regulating autophagy flux. Hence, in this study, we investigated the effects of GTPs on renal function in high-fat diet-fed rats and explored the underlying mechanisms.

## 2. Materials and Methods

### 2.1. Materials

GTPs (purity > 98%) were obtained from Fuzhou Rimian Inc. (Fuzhou, China); EGCG (purity ≥ 95%) and palmitic acid (PA) were purchased from Sigma-Aldrich (St. Louis, MO, USA). Rabbit polyclonal antibodies of Beclin-1, p62/SQSTM and Rabbit monoclonal antibodies of GAPDH, AMP-activated protein kinase (AMPK), *p*-AMPK (Thr172) were purchased from Cell Signaling Technology (Beverly, MA, USA). Rabbit polyclonal LC3-II antibody was provided by Novus (Littleton, CO, USA). Mouse monoclonal lysosomal-associated membrane protein 1 (LAMP-1) antibody was obtained from Santa Cruz Biotechnology (Santa Cruz, CA, USA). Rabbit polyclonal antibodies of cathepsin B and cathepsin D were purchased from Proteintech (Wuhan, China) and mouse monoclonal β-Actin antibody was purchased from Sigma-Aldrich (St. Louis, MO, USA).

### 2.2. Animals

Male Wistar rats (150–160 g) were obtained from Hubei Research Center of Laboratory Animals (Wuhan, China) and housed in well-ventilated cages under adjustable conditions (12 h light/dark cycle, 22–26 °C, 55%–65% humidity) with unrestricted access to water. The animal experiments were approved by The Institutional Animal Care and Use Committee of Huazhong University of Science and Technology (IACUC Number: 412) and the animals were cared for according to guidelines and authorization for care and use of laboratory animals. After one week’s acclimation, they were randomly divided into 6 groups: (1) standard diet *ad libitum* (STD; 20% crude protein, 5% lipids, 4% crude fiber, 8% ash, 1.4% calcium, 0.9% phosphorus, 2% vitamin and 52% carbohydrates, *w/w*); (2) 30% dietary restriction of standard diet (DR); (3) modified high-fat diet *ad libitum* (HFD; 60% standard diet, 12% lard, 10% sugar, 8% yolk powder, 6% peanut powder, 3% casein and 1% milk powder, *w/w* ); (4) STD with 200 mg/kg(bw)/day GTPs (STD+GTPs); (5) DR with 200 mg/kg(bw)/day GTPs (DR+GTPs); and (6) HFD with 200 mg/kg(bw)/day GTPs (HFD+GTPs). Animals on the DR and DR+GTPs groups received 30% less food than standard diet. Animals were maintained on the interventions for 18 weeks. Body weights were measured once a week, food consumption was recorded every day and the quantity of food given to the DR rats was adjusted daily according to the food consumption of the STD rats.

Rats were transferred to metabolic cages for 24-h urine collection. Samples were collected and centrifuged at 1000 g for 10 min. Urine was transferred to tubes and volume was measured. Urinary *N*-acetyl-β-d-glucosaminidase (NAG) (Jiancheng Bioengineering, Nanjing, China) and urinary creatinine (BIOSINO, Beijing, China) were detected using a commercial kit. Prior to sacrifice, rats were fasted overnight and blood was collected, then serum was obtained by centrifugation (1200 g for 15 min at 4 °C). Serum total cholesterol, triglyceride, blood glucose and creatinine concentrations were assayed by the colorimetric method (BIOSINO, Beijing, China). Serum cystatin C (Cys C) was measured using ELISA kits (Biovendor, Heidelberg, Germany). Endogenous creatinine clearance rate (Ccr) (mL/h/kg(bw) = [urinary creatinine (μmol/L) × urine volume (mL/h)]/serum creatinine (μmol/L)/body weight (g). Simultaneously, rat kidneys were rapidly taken out, immediately frozen in liquid nitrogen, and stored at −80 °C until analysis. Kidney coefficient = kidney weight/body weight × 100.

### 2.3. Cell Culture 

Human proximal tubular epithelial cells (HK-2) were kindly provided by Stem Cell Bank, Chinese Academy of Sciences (Shanghai, China). Cells were cultured with DMEM/F12 supplemented with 10% fetal bovine serum and 10 μg/mL gentamicin in a humidified atmosphere containing 5% CO_2_ incubator at 37 °C. All experiments were conducted following 8–15 passages when the cells reached 80%–90% confluence.

### 2.4. Morphologic and Red O Oil Staining 

Tissues were removed and fixed with 4% paraformaldehyde solution, then were embedded in paraffin, sectioned in 5-µm sections and stained with hematoxylin and eosin (H & E). Tissues were embedded in Tissue-Tek OCT compound for red O oil staining, and samples were scanned under a light microscope.

### 2.5. Western Blot Analysis

Rat kidney tissues of the six groups were rapidly homogenized and then lysed in cold RIPA extraction buffer (Beyotime, Haimen, China) and then incubated in ice for 2 h. The lysate was centrifuged at 12000 rpm for 15 min at 4 °C and the supernatant was collected. The protein concentration was determined using BCA Assay (BOSTER, Wuhan, China). Equal amounts of protein solutions were mixed with Sodium dodecylsulfate sample buffer and incubated for 5 min at 98 °C before loading. Sodium dodecyl sulfate-polyacrylamide gel electrophoresis (SDS-PAGE) and immunological blotting were performed on the basis of the method of Amersham Biosciences (Buckinghamshire, UK). Protein expression bands were detected by a chemiluminescent detection system (Syngen, Cambridge, UK) and analyzed with Chemidoc-Quantity-One (Bio-Rad Laboratories) software.

### 2.6. Statistical Analysis

Data were expressed as means ± SD in animal experiments and as means ± SEM in cell experiments. Protein expression experiments were reproduced three to five times, and representative experiments are presented in figures. Statistical analysis was performed by ANOVA using SPSS 13.0 (SPSS, Chicago, IL, USA). All *p* values were two-tailed and differences were considered significant when *p* < 0.05.

## 3. Results

### 3.1. Body Weight, Blood Glucose and Serum Lipid Levels in Different Diet Groups

**Figure 1:**
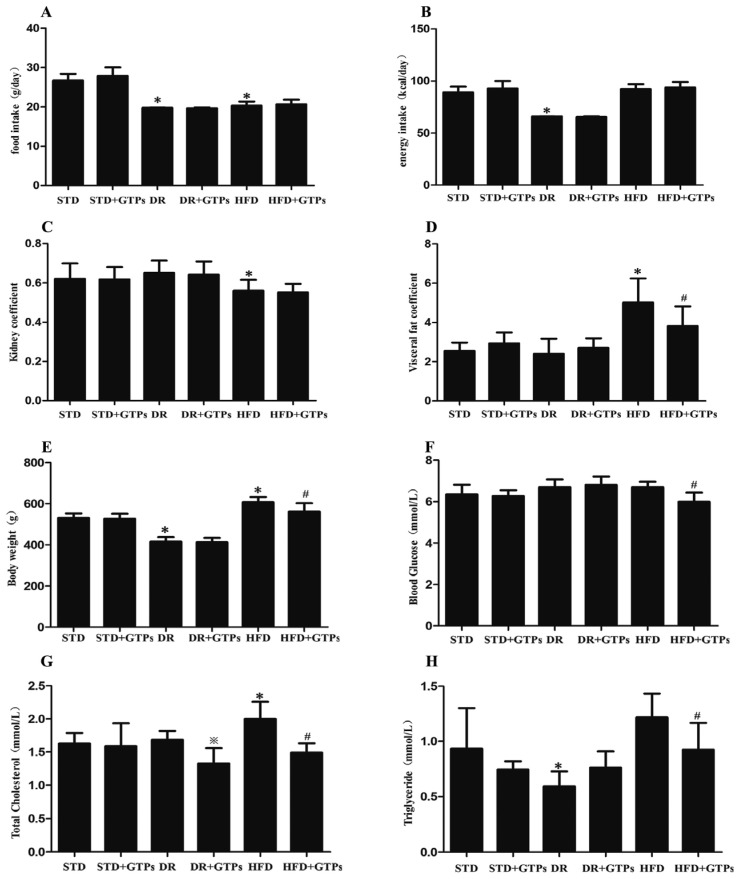
Body weight, total energy intake, blood glucose and serum lipid levels of different diet-treated rats. (**A**) Food intake; (**B**) Total energy intake; (**C**) Kidney coefficient; (**D**) Visceral fat coefficient; (**E**) Body weight; (**F**) Serum blood glucose; (**G**) Serum total cholesterol; and (**H**) Serum triglyceride. Data are expressed as the mean ± SD (*n* = 6–8). * *p* < 0.05 vs. standard diet group (STD); ^#^ *p* < 0.05 vs. high-fat diet group (HFD); ^※^ *p* < 0.05 vs. dietary restriction group (DR).

Compared to the STD group (Figure 1), body weight, visceral fat coefficient, and total cholesterol were markedly increased and kidney coefficient was significantly decreased in HFD, while body weight and triglyceride levels were decreased significantly in DR group (*p* < 0.05). In addition, GTPs improved body weight, visceral fat coefficient and kidney coefficient of HFD-fed rats (*p* < 0.05), and reduced blood glucose, triglyceride, and total cholesterol levels in HFD group (*p* < 0.05), even though there was no significant difference of energy intake between HFD and HFD+GTPs. GTPs reduced total cholesterol levels of DR-fed rats compared to DR group (*p* < 0.05). 

### 3.2. Renal Function in Different Diet Groups

As shown in Table 1, urinary NAG and serum Cys C levels were significantly increased (*p* < 0.05), while no significant change of Ccr was observed in HFD group compared to STD group. However, a significant increase in Ccr and a decrease in urinary NAG was shown in rats fed on DR with or without GTPs compared to STD (*p* < 0.05). No significant differences were observed in the levels of these three biomarkers between STD+GTPs and STD rats. However, HFD+GTPs exhibited lower urinary NAG and serum Cys C than those in HFD group (*p* < 0.05). These results suggested that DR and GTPs have no effects on renal function in healthy rats and treatment with GTPs ameliorated early kidney injury induced by HFD.

### 3.3. Autophagy Activity of Kidney in Different Diet Groups

**Figure 2:**
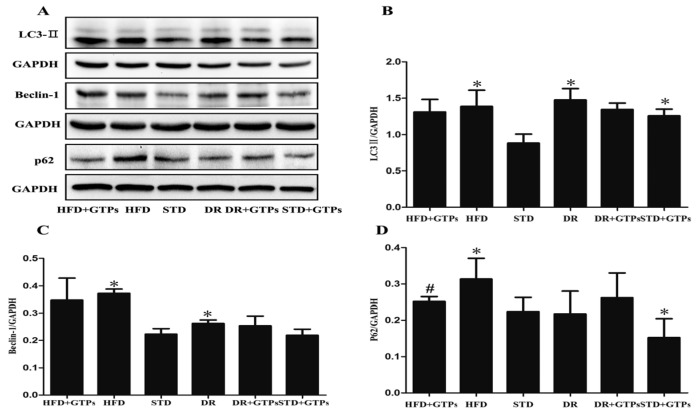
The effects of GTPs on renal autophagy of different diet-treated rats. *(***A***)* Western blot analysis of LC3-II, Beclin-1 and p62; *(***B***)* The relative protein levels of LC3-II; (**C**) Beclin-1 and (**D**) p62. GAPDH served as loading controls. Data are expressed as the mean ± SD (*n* = 3–5) * *p* < 0.05 vs. standard diet group (STD); ^#^ *p* < 0.05 vs. high fat diet group (HFD).

As shown in Figure 2, the protein expressions of LC3-II *(*Figure 2B*),* Beclin-1 (Figure 2C) and p62/SQSTM1 *(*Figure 2D*)* in HFD group were at higher levels than those of STD group (*p* < 0.05). And the protein expression of LC3-II in the kidneys was significantly increased in STD+GTPs, DR and DR+GTPs compared to STD group, and protein expression of Beclin-1 was increased in DR (*p* < 0.05); besides, the expression of p62/SQSTM1 was significantly decreased in STD+GTPs compared to STD (*p* < 0.05). Furthermore, decreased p62/SQSTM1 expression was detected in HFD+GTPs group compared to HFD group (*p* < 0.05). These results revealed that GTPs and DR can induce autophagy in rat kidney and GTPs can mitigate HFD-induced autophagy impairment. 

### 3.4. Lysosome Function of Kidney in Different Diet Groups

**Figure 3:**
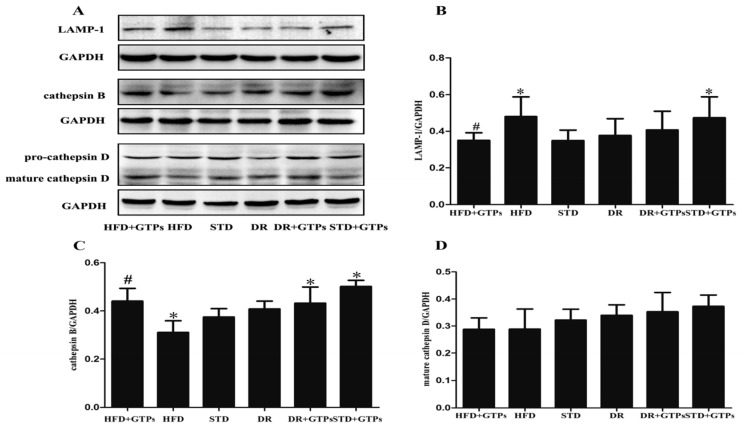
The effects of GTPs on renal lysosome of different diet-treated rats. (**A**) Western blot analysis of renal LAMP-1, cathepsin B and cathepsin D; (**B**) The relative protein levels of renal LAMP-1; (**C**) cathepsin B and (**D**) cathepsin D. GAPDH served as loading controls. Data are expressed as the mean ± SD (*n* = 3–5) * *p* < 0.05 vs. standard diet group (STD); ^#^ *p* < 0.05 vs. high fat diet group (HFD).

HFD significantly increased the expression of LAMP-1 and decreased cathepsin B expression compared to STD group (*p* < 0.05). The protein level of cathepsin B was increased in STD+GTPs and DR+GTPs, and LAMP-1 expression was elevated in STD+GTPs compared to STD (*p* < 0.05). Moreover, GTPs reduced the expression of LAMP-1 and enhanced the expression of cathepsin B in HFD-fed rats (*p* < 0.05*,* Figure 3B,C). No significant differences in the expression of cathepsin D were observed in all groups (Figure 3D). All together, these results demonstrated that HFD caused lysosomal hydrolases reduction and lysosome overloading, which can be improved by GTPs.

### 3.5. EGCG Enhanced Autophagic Flux and Ameliorated PA-Induced Autophagy Impairment in HK-2 Cells

**Figure 4:**
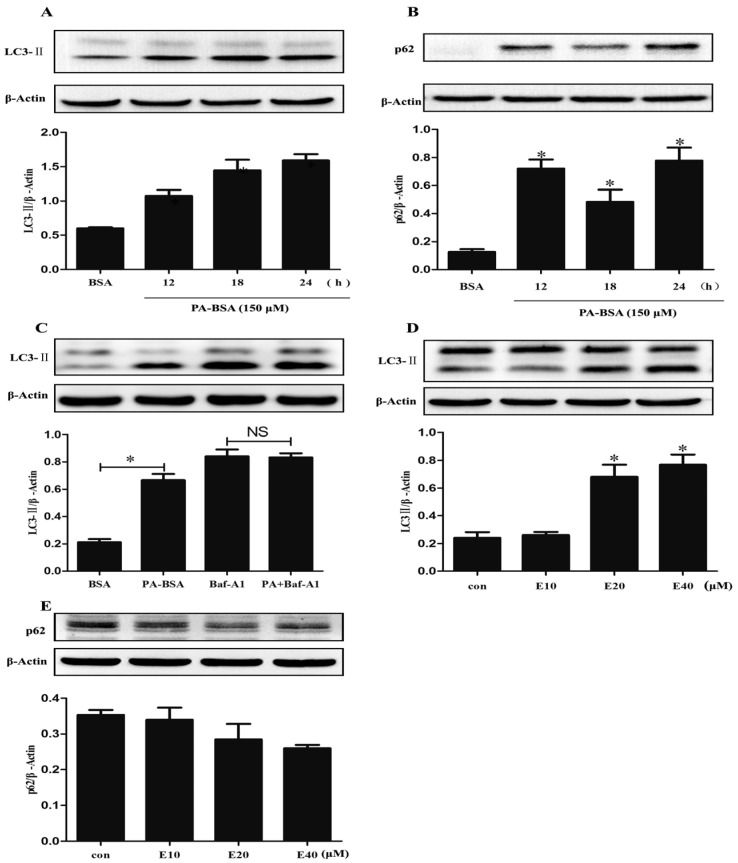
The effects of EGCG and PA-BSA on autophagy in HK-2 cells. The relative protein expression of (**A**) LC3-II and (**B**) p62 in HK-2 cells exposed to PA-BSA (150 μM); (**C**) LC3-II turnover; HK-2 cells were pretreated with bafilomycin A1 (100 nM) for 3 h, subsequently incubated with PA-BSA (150 μM) for 12 h and LC3-II level was evaluated by western blot analysis. The relative protein levels of (**D**) LC3-II and (**E**) p62 in HK-2 cells exposed to EGCG. β-Actin served as loading controls. Data are expressed as mean ± SEM (*n* = 3–5). * *p* < 0.05 vs. control group (BSA).

**Figure 5:**
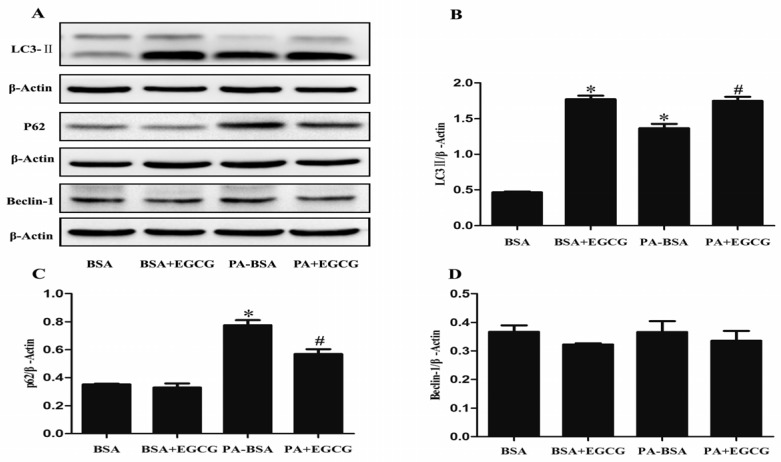
EGCG ameliorates PA-BSA induced autophagic flux impairment in HK-2 cells. Western blot analysis of LC3-II, Beclin-1 and p62 in HK-2 cells pretreated with 40 μM EGCG for 24 h and subsequently incubated with PA-BSA (150 μM) for 12 h. (**A**) Western blot analysis of LC3-II, Beclin-1 and p62; (**B**) The relative protein levels of LC3-II; (**C**) Beclin-1 and (**D**) p62. β-Actin served as loading controls. Data are expressed as mean ± SEM (*n* = 3–5).* *p* < 0.05 vs. control group (BSA); ^#^ *p* < 0.05 vs. PA-BSA group.

PA-BSA treatment increased the protein expression of LC3-II and p62/SQSTM1 in HK-2 cells (Figure 4A,B). LC3-II turnover was further examined in the presence and absence of bafilomycin A1. Pretreatment with bafilomycin A1 (100 nM) increased LC3-II accumulation; PA did not further increase LC3-II accumulation when treated with bafilomycin A1 (Figure 4C). EGCG increased LC3-II expression in a dose-dependent manner (Figure 4D) while it did not alter p62/SQSTM1 expression in HK-2 cells (Figure 4E). Co-treatment with EGCG increased expression of LC3-II *(*Figure 5B*)* and reduced the expression of p62/SQSTM1 compared to PA-BSA treatment alone (Figure 5C*)*. These results showed that EGCG enhanced autophagic flux and EGCG ameliorated PA-induced autophagy impairment in HK-2 cells.

### 3.6. GTPs and EGCG Stimulated AMPK Activity In Vivo and In Vitro, Respectively

**Figure 6:**
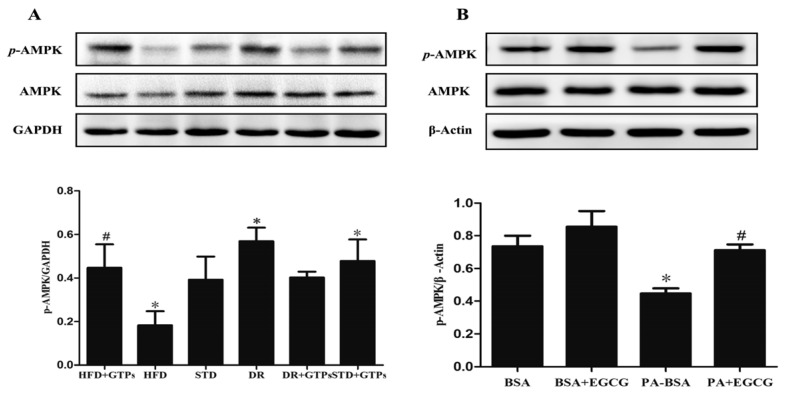
Effect of GTPs or EGCG on AMPK activation. (**A**) The relative protein expression of *p*-AMPK (Thr172) and total AMPK in the kidney of different diet-treated rats (**B**) and in HK-2 cells pretreated with 40 μM EGCG for 24 h and subsequently incubated with PA-BSA (150 μM) for 12 h were measured by western blot. GAPDH and β-Actin served as loading controls in rats and HK-2 cells respectively. Data are expressed as mean ± SD in rats or mean ± SEM in HK-2 cells (*n* = 3–5). * *p* < 0.05 vs. ad standard diet (STD) or control group (BSA); ^#^ *p* < 0.05 vs. high-fat diet group (HFD) or PA-BSA group.

Phosphorylation of AMPK was significantly decreased in HFD, while it increased in DR and STD+GTPs compared to STD group. Nevertheless, GTPs significantly increased the expression of AMPK phosphorylation in the kidney of HFD rats (Figure 6A). In HK-2 cells, PA-BSA induced a decrease in phosphorylation of AMPK, which was improved by EGCG (Figure 6B). Altogether, GTP treatment attenuated HFD-induced autophagy suppression partially via AMPK pathway.

## 4. Discussion

In this study, we showed that HFD rats exhibited an increased body weight gain, fat accumulation, serum blood glucose and lipid levels and liver lipid storage. Ccr can represent the glomerular filtration function to some extent, but it usually changes in serious renal damage. Serum Cys C is considered a biomarker of early renal damage. Urinary NAG is a biomarker in renal tubule injury. The rats fed on HFD also showed increased serum Cys C and urinary NAG activity without altering Ccr and kidney structure, indicating that the renal injury induced by HFD was in early stage. Similar effects have also been observed in mice kidney induced by HFD [28]. The renal benefits of DR have been shown on rats with obesity-linked renal disease [8]. In our study, GTP intervention alleviated HFD-induced renal injury, indicated by reduced serum Cys C levels and urinary NAG activity. Besides, Ccr was increased in DR and DR+GTPs, indicating hyperfiltration. Green tea has been reported to improve kidney function in streptozotocin (STZ)-diabetic rats [25]. Pan and colleagues demonstrated that resveratrol, another kind of phyto-polyphenol, ameliorates renal injury and proteinuria in obese rats induced by a 12-week HFD [29]. Therefore, the results demonstrated that GTPs have protective effects on renal injury induced by HFD.

The autophagy–lysosomal system plays a pivotal role in the homeostasis and survival of renal cells, and dysregulation of autophagy might contribute to the development of kidney diseases [16,30]. There has been extensive work in kidney diabetic models, as in high glucose-cultured proximal tubular epithelial cells (PTECs), in which autophagy activity is increased and serves as a renal protective response. Hence, autophagy activators can attenuate high glucose-induced lipid accumulation [31]. Liu reported that autophagy inactivation is induced by lysosomal impairment in PTECs exposed to advanced glycation end products (AGEs), which results in the accumulation of abnormal proteins [32]. However, little work has been done with HFD and autophagy in the kidney. In this study, increased expression of LC3-II was observed in HFD, DR and STD+GTPs groups, and increased protein expression of Beclin-1 was observed in HFD and DR groups, which indicated increased autophagosomes in these groups. On the contrary, p62/SQSTM1 as an indicator of autophagosome degradation also increased in HFD group, while GTPs decreased p62/SQSTM1 expression. The increase in p62/SQSTM1 protein levels in HFD group may be due to impaired autophagosome degradation. Besides, cathepsin B, the main kidney cysteine protease, was decreased in HFD group and was increased in STD+GTPs group. These results revealed that HFD suppressed autophagy activity and consequently induced LC3-II accumulation, and DR or GTPs enhanced autophagy activity. The hyperfiltration in DR and DR+GTPs might be associated with the autophagy enhancement. Kitada et al. reported that DR promoted autophagy and increased Ccr in diabetic Wistar fatty (fa/fa) rats [18]. The exact explanation needs further investigation. In vitro, increased protein expressions of LC3-II and p62/SQSTM1 were also observed in HK-2 cells exposed to PA-BSA. An accurate examination of LC3-II turnover was the principal method for monitoring autophagic flux. Compared with treatment with bafilomycin A1 alone, PA did not increase the amount of LC3-II when pretreated with bafilomycin A1. Bafilomycin A1 was able to block the fusion of autophagosomes with lysosomes, and the results indicated that the initiation of autophagosomes was not increased; therefore, the increased LC3-II was due to decreased degradation, suggesting PA impaired autophagy flux in HK-2 cells. Taken together, HFD impaired autophagy activity in rat kidney, and the impairment of autophagy may be a causative to renal injury induced by HFD.

Our previous study had demonstrated that GTPs promote autophagy flux and alleviate high glucose-induced inhibition of autophagy in endothelial cells [27]. Similarly, we have shown that DR or GTPs enhanced autophagy activity in kidney of rats. Then we investigated whether GTPs can ameliorate high-fat diet-induced autophagy impairment. In the present study, our results showed that GTP treatment decreased the expression of p62/SQSTM1 in HFD rats, indicating GTPs mitigated autophagy impairment induced by HFD. EGCG is the major constituent of GTPs, and most of the health-promoting effects of GTPs are attributed to EGCG. In vitro, the effects of EGCG on autophagy impairment induced by PA are in accordance with our in vivo study. Moreover, GTP treatment mitigated HFD-induced overloading of lysosome and improved the lysosome function with an increase of cathepsin B expression, suggesting GTPs may promote autophagy through improving lysosome protease. A study reported EGCG has the ability to protect the lysosomal membrane and maintain lysosomal enzyme activity in heart [33]. CR has been reported to enhance autophagy and ameliorate oxidative damage in the heart and kidney of aged rats [34,35]. And, several reports have suggested that some of the beneficial effects of GTPs may be mediated by autophagy [26,36,37,38]. Kim reported that EGCG treatment facilitated inhibition of the fusion process of autophagosome and lysosome induced by palmitate in endothelial cells [38]. EGCG was also shown to stimulate hepatic autophagy by promoting the formation of autophagosomes and increasing lysosomal acidification [37]. Combined with these results, GTPs show similar effects on autophagy enhancement to DR and alleviate HFD-induced autophagy impairment. 

AMPK is a ubiquitous heterotrimeric kinase and is abundantly expressed in the kidney. As a cellular energy sensor, AMPK is one of major regulators of autophagy activity. In addition, AMPK plays an important role in several renal diseases [5,39]. We suppose AMPK may be involved in the protective effects of GTPs against kidney injury induced by HFD. In the present study, DR and GTPs stimulated AMPK activity, while HFD reduced phosphorylation of AMPK. Likewise, GTPs (or EGCG) treatment elevated phosphorylation of AMPK both in the kidney of rats fed on HFD and in HK-2 cells exposed to PA. AMPK activity was reported suppressed in the kidney of mice fed with high-fat diet, and AMPK activation normalized the changes induced by high-fat diet [5,40]. Watanabe et al. demonstrated that DR prevents postinfarction heart failure via enhancing autophagy, and AMPK activation is one possible way to improve autophagic flux through DR [41]. In endothelial cells, EGCG reduced palmitate-induced lipid droplet storage through inducing autophagy via a Ca2+/CaMKKβ/AMPK-mediated mechanism [38]. And resveratrol promoted autophagy via upregulating SIRT3 expression and AMPK phosphorylation in macrophages [42]. Therefore, AMPK may play a role in the autophagy-inducing properties of GTPs in renal protection, which is similar to that of DR. However, the exact mechanism by which GTPs can induce autophagy and alleviate HFD-induced autophagy–lysosome impairment in rat kidney will need further investigation. 

In summary, HFD was shown to suppress AMPK activity, cause lysosome dysfunction, and deteriorate autophagy in kidney of rats, which may contribute to renal injury induced by HFD. GTPs mimic the effects of DR on AMPK stimulation and autophagy enhancement, and ameliorate HFD-induced renal injury and autophagy impairment. As dietary polyphenols including GTPs possess beneficial effects similar to those of DR, it is possible that people keep in good health and enjoy delicious meals at the same time. Therefore, our results suggest GTPs might be a potential renal protective supplement in the Western lifestyle. However, the exact effects of GTPs on human renal protection still need further clinical investigation.

## Figures and Tables

**Table 1:** Effects of GTPs on the kidney function of rats in different diet-treated groups.

Groups	Ccr (mL/h/kg(bw))	Serum Cys C (μg/mL)	Urinary NAG (U/L)
STD	68.35 ± 26.11	1.42 ± 0.39	20.51 ± 4.03
STD+GTPs	61.66 ± 20.69	1.51 ± 0.50	19.35 ± 6.07
DR	109.03 ± 33.02 *	1.34 ± 0.10	13.84 ± 5.28 *
DR+GTPs	102.32 ± 43.40	1.45 ± 0.35	13.74 ± 4.90
HFD	46.77 ± 28.76	1.99 ± 0.34 *	28.54 ± 5.13 *
HFD+GTPs	52.05 ± 18.35	1.45 ± 0.49 ^#^	22.19 ± 2.91 ^#^

* *p* < 0.05 vs. standard diet group (STD); ^#^ *p* < 0.05 vs. high fat diet group (HFD).

## References

[1] Zhang L., Wang F., Wang L., Wang W., Liu B., Liu J., Chen M., He Q., Liao Y., Yu X. (2012). Prevalence of chronic kidney disease in china: A cross-sectional survey. Lancet.

[2] Odermatt A. (2011). The western-style diet: A major risk factor for impaired kidney function and chronic kidney disease. Am. J. Physiol. Renal. Physiol..

[3] Lin J., Fung T.T., Hu F.B., Curhan G.C. (2011). Association of dietary patterns with albuminuria and kidney function decline in older white women: A subgroup analysis from the nurses’ health study. Am. J. Kidney Dis..

[4] Chen J., Wildman R.P., Gu D., Kusek J.W., Spruill M., Reynolds K., Liu D., Hamm L.L., Whelton P.K., He J. (2005). Prevalence of decreased kidney function in Chinese adults aged 35 to 74 years. Kidney Int..

[5] Decleves A.E., Mathew A.V., Cunard R., Sharma K. (2011). Ampk mediates the initiation of kidney disease induced by a high-fat diet. J. Am. Soc. Nephrol..

[6] Zhang R., Yu Y., Deng J., Zhang C., Zhang J., Cheng Y., Luo X., Han B., Yang H. (2016). Sesamin ameliorates high-fat diet–induced dyslipidemia and kidney injury by reducing oxidative stress. Nutrients.

[7] Aydin C., Ince E., Koparan S., Cangul I.T., Naziroglu M., Ak F. (2007). Protective effects of long term dietary restriction on swimming exercise-induced oxidative stress in the liver, heart and kidney of rat. Cell Biochem. Funct..

[8] Maddox D.A., Alavi F.K., Santella R.N., Zawada E.T. (2002). Prevention of obesity-linked renal disease: Age-dependent effects of dietary food restriction. Kidney Int..

[9] Kapahi P., Kaeberlein M., Hansen M. (2016). Dietary restriction and lifespan: Lessons from invertebrate models. Ageing Res. Rev..

[10] Wang Z., Choi M.E. (2014). Autophagy in kidney health and disease. Antioxid. Redox Signal..

[11] De Rechter S., Decuypere J.P., Ivanova E., van den Heuvel L.P., De Smedt H., Levtchenko E., Mekahli D. (2016). Autophagy in renal diseases. Pediatr. Nephrol..

[12] Yamahara K., Kume S., Koya D., Tanaka Y., Morita Y., Chin-Kanasaki M., Araki H., Isshiki K., Araki S., Haneda M. (2013). Obesity-mediated autophagy insufficiency exacerbates proteinuria-induced tubulointerstitial lesions. J. Am. Soc. Nephrol..

[13] Wang X., Liu J., Zhen J., Zhang C., Wan Q., Liu G., Wei X., Zhang Y., Wang Z., Han H. (2014). Histone deacetylase 4 selectively contributes to podocyte injury in diabetic nephropathy. Kidney Int..

[14] Jung M., Lee J., Seo H.Y., Lim J.S., Kim E.K. (2015). Cathepsin inhibition-induced lysosomal dysfunction enhances pancreatic beta-cell apoptosis in high glucose. PLoS ONE.

[15] Lamore S.D., Wondrak G.T. (2012). Autophagic-lysosomal dysregulation downstream of cathepsin b inactivation in human skin fibroblasts exposed to uva. Photochem. Photobiol. Sci..

[16] Repnik U., Stoka V., Turk V., Turk B. (2012). Lysosomes and lysosomal cathepsins in cell death. Biochim. Biophys. Acta.

[17] Kanamori H., Takemura G., Maruyama R., Goto K., Tsujimoto A., Ogino A., Li L., Kawamura I., Takeyama T., Kawaguchi T. (2009). Functional significance and morphological characterization of starvation-induced autophagy in the adult heart. Am. J. Pathol..

[18] Kitada M., Takeda A., Nagai T., Ito H., Kanasaki K., Koya D. (2011). Dietary restriction ameliorates diabetic nephropathy through anti-inflammatory effects and regulation of the autophagy via restoration of sirt1 in diabetic wistar fatty (fa/fa) rats: A model of type 2 diabetes. Exp. Diabetes Res..

[19] Lambert J.D. (2013). Does tea prevent cancer? Evidence from laboratory and human intervention studies. Am. J. Clin. Nutr..

[20] Amiot M.J., Riva C., Vinet A. (2016). Effects of dietary polyphenols on metabolic syndrome features in humans: A systematic review. Obes. Rev..

[21] Cong L., Cao C., Cheng Y., Qin X.Y. (2016). Green tea polyphenols attenuated glutamate excitotoxicity via antioxidative and antiapoptotic pathway in the primary cultured cortical neurons. Oxid. Med. Cell. Longev..

[22] Li Y.W., Zhang Y., Zhang L., Li X., Yu J.B., Zhang H.T., Tan B.B., Jiang L.H., Wang Y.X., Liang Y. (2014). Protective effect of tea polyphenols on renal ischemia/reperfusion injury via suppressing the activation of tlr4/nf-kappab p65 signal pathway. Gene.

[23] Kuriyama S., Shimazu T., Ohmori K., Kikuchi N., Nakaya N., Nishino Y., Tsubono Y., Tsuji I. (2006). Green tea consumption and mortality due to cardiovascular disease, cancer, and all causes in japan: The ohsaki study. JAMA.

[24] Liu J., Liu S., Zhou H., Hanson T., Yang L., Chen Z., Zhou M. (2016). Association of green tea consumption with mortality from all-cause, cardiovascular disease and cancer in a Chinese cohort of 165,000 adult men. Eur. J. Epidemiol..

[25] Renno W.M., Abdeen S., Alkhalaf M., Asfar S. (2008). Effect of green tea on kidney tubules of diabetic rats. Br. J. Nutr..

[26] Lee J.H., Moon J.H., Kim S.W., Jeong J.K., Nazim U.M., Lee Y.J., Seol J.W., Park S.Y. (2015). Egcg-mediated autophagy flux has a neuroprotection effect via a class iii histone deacetylase in primary neuron cells. Oncotarget.

[27] Zhang P.W., Tian C., Xu F.Y., Chen Z., Burnside R., Yi W.J., Xiang S.Y., Xie X., Wu N.N., Yang H. (2016). Green tea polyphenols alleviate autophagy inhibition induced by high glucose in endothelial cells. Biomed. Environ. Sci..

[28] Tikoo K., Sharma E., Amara V.R., Pamulapati H., Dhawale V.S. (2016). Metformin improves metabolic memory in high fat diet (hfd)-induced renal dysfunction. J. Biol. Chem..

[29] Pan Q.R., Ren Y.L., Zhu J.J., Hu Y.J., Zheng J.S., Fan H., Xu Y., Wang G., Liu W.X. (2014). Resveratrol increases nephrin and podocin expression and alleviates renal damage in rats fed a high-fat diet. Nutrients.

[30] Yamamoto-Nonaka K., Koike M., Asanuma K., Takagi M., Oliva Trejo J.A., Seki T., Hidaka T., Ichimura K., Sakai T., Tada N. (2016). Cathepsin d in podocytes is important in the pathogenesis of proteinuria and ckd. J. Am. Soc. Nephrol..

[31] Xu Y., Liu L., Xin W., Zhao X., Chen L., Zhen J., Wan Q. (2015). The renoprotective role of autophagy activation in proximal tubular epithelial cells in diabetic nephropathy. J. Diabetes Complicat..

[32] Liu W.J., Shen T.T., Chen R.H., Wu H.L., Wang Y.J., Deng J.K., Chen Q.H., Pan Q., Huang Fu C.M., Tao J.L. (2015). Autophagy-lysosome pathway in renal tubular epithelial cells is disrupted by advanced glycation end products in diabetic nephropathy. J. Biol. Chem..

[33] Devika P.T., Prince P.S. (2008). Preventive effect of (-)epigallocatechin-gallate (egcg) on lysosomal enzymes in heart and subcellular fractions in isoproterenol-induced myocardial infarcted wistar rats. Chem. Biol. Interact..

[34] Wohlgemuth S.E., Julian D., Akin D.E., Fried J., Toscano K., Leeuwenburgh C., Dunn W.A. (2007). Autophagy in the heart and liver during normal aging and calorie restriction. Rejuvenation Res..

[35] Ning Y.C., Cai G.Y., Zhuo L., Gao J.J., Dong D., Cui S., Feng Z., Shi S.Z., Bai X.Y., Sun X.F. (2013). Short-term calorie restriction protects against renal senescence of aged rats by increasing autophagic activity and reducing oxidative damage. Mech. Ageing Dev..

[36] Kim H.S., Quon M.J., Kim J.A. (2014). New insights into the mechanisms of polyphenols beyond antioxidant properties; lessons from the green tea polyphenol, epigallocatechin 3-gallate. Redox Biol..

[37] Zhou J., Farah B.L., Sinha R.A., Wu Y., Singh B.K., Bay B.H., Yang C.S., Yen P.M. (2014). Epigallocatechin-3-gallate (egcg), a green tea polyphenol, stimulates hepatic autophagy and lipid clearance. PLoS ONE.

[38] Kim H.S., Montana V., Jang H.J., Parpura V., Kim J.A. (2013). Epigallocatechin gallate (egcg) stimulates autophagy in vascular endothelial cells: A potential role for reducing lipid accumulation. J. Biol. Chem..

[39] Sharma K. (2016). Obesity and diabetic kidney disease: Role of oxidant stress and redox balance. Antioxid. Redox Signal.

[40] Declèves A.-E., Zolkipli Z., Satriano J., Wang L., Nakayama T., Rogac M., Le T.P., Nortier J.L., Farquhar M.G., Naviaux R.K. (2014). Regulation of lipid accumulation by amk-activated kinase in high fat diet–induced kidney injury. Kidney Int..

[41] Watanabe T., Takemura G., Kanamori H., Goto K., Tsujimoto A., Okada H., Kawamura I., Ogino A., Takeyama T., Kawaguchi T. (2014). Restriction of food intake prevents postinfarction heart failure by enhancing autophagy in the surviving cardiomyocytes. Am. J. Pathol..

[42] Duan W.J., Li Y.F., Liu F.L., Deng J., Wu Y.P., Yuan W.L., Tsoi B., Chen J.L., Wang Q., Cai S.H. (2016). A sirt3/ampk/autophagy network orchestrates the protective effects of trans-resveratrol in stressed peritoneal macrophages and raw 264.7 macrophages. Free Radic. Biol. Med..

